# SLE Antibody-Secreting Cells Are Characterized by Enhanced Peripheral Maturation and Survival Programs

**DOI:** 10.21203/rs.3.rs-3016327/v1

**Published:** 2023-06-27

**Authors:** Weirong Chen, So-Hee Hong, Scott A. Jenks, Fabliha A. Anam, Christopher M. Tipton, Matthew C. Woodruff, Jennifer R. Hom, Kevin S. Cashman, Caterina Elisa Faliti, Xiaoqian Wang, Shuya Kyu, Chungwen Wei, Christopher D. Scharer, Tian Mi, Sakeenah Hicks, Louise Hartson, Doan C. Nguyen, Arezou Khosroshahi, Saeyun Lee, Youliang Wang, Regina Bugrovsky, Yusho Ishii, F. Eun-Hyung Lee, Ignacio Sanz

**Affiliations:** 1 Department of Medicine, Division of Rheumatology, Lowance Center for Human Immunology, School of Medicine, Emory University, Atlanta, GA, USA; 2 Department of Medicine, Division of Pulmonary, Allergy, Critical Care and Sleep Medicine, School of Medicine, Emory University, Atlanta, GA, USA; 3 Department of Microbiology and Immunology, School of Medicine, Emory University, Atlanta, GA, USA

## Abstract

Systemic Lupus Erythematosus (SLE) is an autoimmune disease characterized by multiple autoantibodies, some of which are present in high titers in a sustained, B cell-independent fashion consistent with their generation from long-lived plasma cells (LLPC). Active SLE displays high numbers of circulating antibody-secreting cells (ASC). Understanding the mechanisms of generation and survival of SLE ASC would contribute important insight into disease pathogenesis and novel targeted therapies. We studied the properties of SLE ASC through a systematic analysis of their phenotypic, molecular, structural, and functional features. Our results indicate that in active SLE, relative to healthy post-immunization responses, blood ASC contain a much larger fraction of newly generated mature CD19^−^ CD138^+^ ASC similar to bone marrow (BM) LLPC. SLE ASC were characterized by morphological and structural features of premature maturation. Additionally, SLE ASC express high levels of CXCR4 and CD138, and molecular programs consistent with increased longevity based on pro-survival and attenuated pro-apoptotic pathways. Notably, SLE ASC demonstrate autocrine production of APRIL and IL-10 and experience prolonged in vitro survival. Combined, our findings indicate that SLE ASC are endowed with enhanced peripheral maturation, survival and BM homing potential suggesting that these features likely underlie BM expansion of autoreactive PC.

## Introduction

Plasma cells (PC) represent a critical effector immune cell type responsible for both protective and pathogenic antibody responses. Despite being often lumped together, PC exhibit significant diversity in terms of their characteristics, including homeostasis, phenotype, location and longevity^1^. The PC compartment can be envisioned as a heterogeneous collection of antibody-secreting cell populations (ASC) that share essential properties, including constitutive antibody secretion, morphology, expression of licensing transcription factors (TF), such as Blimp-1, XBP1, and IRF4, silencing of PAX5, and in humans, high levels of CD38 and CD27 expression with or without CD19^1^. ASC sharing these identifiers can be differentiated into cycling (Ki-67^+^) plasmablasts (PB), which burst into the circulation approximately seven days after acute antigenic stimulation and undergo apoptosis within two weeks (short-lived PB), and resting plasma cells (PC), which can persist for years in the BM in mice even without stimulating antigen^2^. Our previous research demonstrated that human LLPC, responsible for maintaining antibodies against measles and mumps for decades after infection, reside within a CD19^−^ CD138^+^ PC population in the human BM^1^.

SLE is a systemic autoimmune disease in which defective B cell tolerance leads to the production of large amounts of pathogenic autoantibodies, some of which persist throughout the disease with stable serum titers even after sustained depletion of precursor B cells. These long-lived autoantibodies include anti-RNA binding proteins (RBP), such as Smith/RNP, and anti-Ro. In contrast, other SLE autoantibodies, including anti-dsDNA, anti-ribosomal P, and 9G4 antibodies, fluctuate with disease activity^3^ and immunosuppressive therapy^4, 5^. The behavior of persistent serum autoantibodies can be explained by the accumulation of B cell-independent BM LLPC. In addition, local PC are a major contributor to the pathogenesis and outcome of lupus nephritis (LN), a critical disease manifestation. Despite their central pathogenic role and a growing understanding in animal models, the biology of PC in human SLE is still poorly understood and understudied. Therefore, our ability to therapeutically modulate PC in this disease is quite limited. This is a critical unmet need, as B cell depletion can only impede the new generation of PC while failing to eliminate pre-formed pathogenic LLPC.

To address these important knowledge gaps, we undertook a systematic analysis of peripheral blood ASC in patients with SLE, and compared their phenotypic and morphological characteristics, molecular regulatory programs, and survival properties with those of their normal counterparts generated in healthy adults following immunization. Our results reveal that diverse SLE ASC populations exhibit enhanced peripheral maturation originating from shared precursors, increased expression of the CXCR4 and prolonged survival, at least partially attributed to the regulation of apoptotic programs. Furthermore, SLE ASC display elevated levels of CD138 and autocrine production of APRIL and IL-10. Together, our results suggest that SLE ASC possess enhanced peripheral maturation, survival and BM homing potential, and these distinctive features may contribute to the expansion of autoreactive PC within the SLE BM.

This study sheds light on the mechanisms underlying the accumulation of autoreactive long-lived SLE PC, providing novel and valuable insights that could pave the way for the development of PC-targeted therapies. Such advancements in understanding have the potential for rational application across various disease segments, offering avenues for improved treatment strategies.

## Methods

### Human Subjects

All research was approved by the Emory University Institutional Review Board (Emory IRB numbers IRB00058515 and IRB00057983) and was performed in accordance with all relevant guidelines and regulations. Written informed consent was obtained from all participants. Healthy donors (n = 45) were recruited using promotional materials approved by the Emory University Institutional Review Board. Healthy subjects received the influenza vaccinations (n = 24) as part of routine medical care. Subjects with systemic lupus erythematosus (n = 176) were recruited from Emory University Hospital and Grady Hospital in Atlanta, GA, USA. Peripheral blood mononuclear cells were isolated on days 6–7 after vaccination for all vaccinated subjects. SLE patients fulfilled four or more criteria of the modified American College of Rheumatology classification (http://www.rheumatology.org/Practice/Clinical/Indexes/Systemic_Lupus_Erythematosus_Disease_Activity_Index_SELENA_Modification/) and were routinely evaluated by expert rheumatologists at the Emory University Hospital and Grady Hospital. Moderate-to-severe flare were classified according to the flare index of Safety of Estrogen in Lupus: National Assessment-Systemic Lupus Erythematosus Disease Activity Index (SELENA-SLEDAI).

### Multi-color Flow Cytometry and Sorting

Cells were isolated from peripheral blood using Ficoll density gradient centrifugation and perioheral blood mononuclear cells (PBMC) were stained with the following anti-human antibody staining reagents: CD3-BV711 (clone: HIT3a, BD Biosciences); CD3-PE-Cy5.5 (clone: 7D6, Fisher Scientific); CD14-BV711 (clone: M5E2, BD Biosciences); CD14-PE-Cy5.5 (clone: TuK4, Thermo Fisher Scientific); IgD-FITC (IA6-2, BD Biosciences); CD19-PE-Cy7(clone: SJ25C1, BD Biosciences); CD27-APC-eFluor780 (clone: O323, Fisher Scientific); CD38-V450 (clone: HIT2, BD Biosciences); CD138-APC (clone: 44F9, Miltenyi Biotec); CXCR4-PE (clone: 12G5, BioLegend); CXCR4-BV711 (clone:12G5, BD Biosciences); CXCR3-PE(G025H7, BioLegend); Blimp1-PE (6D3, BD Biosciences); BCMA-PE (19F2, BioLegend), IL-6R-PE (M5, BD Biosciences). Approximately, 1 × 10^3^ to 5 × 10^3^ were collected for each cell population by using FACSAria II (BD Biosciences).

### AIRR-Seq

Sorted ASC populations (Pop 2, Pop 3, Pop 4 and Pop 5) from active SLE patients were used to extract the total cellular RNA by using the RNeasy Mini Kit (Qiagen, Inc. Valencia, CA) according to the manufacturer’s protocol. Approximately 400 pg of RNA was subjected to reverse transcription using the iScript RT kit (BioRad, Inc., Hercules, CA). Resulting cDNA products were combined with 50 nM VH1-VH6 specific primers and 250 nM Ca, Cm, and Cg specific primers in a 25 μl PCR reaction using High Fidelity Platinum PCR Supermix (Life Technologies, Carlsbad, CA). Nextera indices were added, and products were sequenced on an Illumina MiSeq with a depth of approximately 50,000 sequences per sample. Sequences were quality filtered and aligned with IMGT.org/HighV-quest. Sequences were then analyzed for V region mutations and clonality using programs developed in-house and made previously available for public use. All clonal assignments were based on matching V and J regions, matching CDR3 length, and 85% CDR3 homology. Custom scripts and in-house developed analysis software were used with R, Matlab or Circos tools for visualization^6^.

### In vitro plasma cell cultures

In vitro plasma cell cultures were performed as previously described by our lab^7^. Sorted ASC populations from active SLE patients and post-vaccination healthy donors were cultured in the secretome from BM-derived mesenchymal stromal cells in 96-well U-shaped bottom cell culture plates in 37 °C in a humid, 5% CO_2_, 95% air (20% O_2)_ incubator for designated days. Cells were harvest for IgG Elispot to assess ASC survival. Exogenous factors including APRIL blocking antibody (human APRIL Antibody, Bio-techne), IL-10 blocking antibody (human IL-10 Antibody, Bio-techne), and their corresponding isotype controls (Monoclonal Mouse IgG_1_ and Monoclonal Mouse IgG_2B_, Bio-techne) were added to cultures at day 0 post cell sorting, and their concentrations were titrated and optimized based on manufacture’s recommendations. Following the incubation, supernatant was collected for ELISA and cells were harvested for ELISPOT assays.

### Total IgG ELISPOT assay

Total IgG ELISPOT assay was performed as previously described by our lab^7^. Sorted ASC populations were added to 96-well ELISPOT plates (MAIPS4510 96 well) pre-coated with anti-human IgG (5 μg/ml, Jackson Immunoresearch). After overnight incubation, weels were washed, and bound antibodies were detected with alkaline phosphatase-conjugated anti-human IgG antibody (1 μg/ml) and developed with VECTOR Blue Alkaline Phosphatase Substrate Kit III (Vector Laboratories). Spots in each well were counted using the CTL immunospot reader (Cellular Technologies Ltd). Results were expressed as the ratio of antigen-specific spots/total IgG spots.

### Enzyme-linked immunosorbent assay (ELISA)

ELISA assay was performed as previously described by our lab^8^. Costar assay high-binding plates were coated with 4 ug/ml ELISA capture antibody IL-10 (Human IL-10 Antibody, Bio-techne) or APRIL (Human APRIL Antibody, Bio-techne) overnight, and then washed with PBS with 0.1% Tween-20 and blocked with SuperBlock blocking buffer for 45 minutes. Plates were next incubated with culture supernatants for 60 minutes. Bound antibodies were then detected with paired detection secondary antibodies at 1.5 ug/ml followed by chromogenic detection with KPL BluePhos Microwell Phosphatase Substrate Kit.

### RNA sequencing and analysis

RNA sequencing was performed as previously described by our lab^9^. Populations including Pop 2 (CD19+ CD138−), Pop 3 (CD19+ CD138+), Pop 5 (CD19− CD138+), and naïve B cells (CD19+ IgD+ CD27−), isolated from active SLE patients or influenza-vaccinated healthy subjects were sorted directly into lysis buffer. RNA was isolated using the AllPrep DNA/RNA Mini kit (Qiagen) according to the manufacturer’s instruction. Total RNA (50pg) was used as input for the ten cycles of PCR amplification by using SMART-seq v3 cDNA synthesis kit (Takara). Libraries were quantified by qPCR and size distribution was examined by Bioanalyer before pooling and sequencing on Illumina HiSeq 2500 sequencer using 50 base pair paired-end sequencing.

### RNA sequencing data analysis

RNA sequencing data analysis was performed as previously described in our lab^9, 10^. Raw fastq files were mapped to the human genome using TopHat2 v.2.0.13 with the hg19 version and the UCSC KnownGene reference transcriptome. All duplicate reads were excluded with PICARD v.1.127, followed by quantification with Partek Flow Genomics Suite (Partek Inc.) using the union model in HTSeq with the default setting, and library-size normalization using the DESeq2 with default parameters. Differential gene expression analysis was determined with a generalized linear effect model. For disease differences covariates included cell type and patients; for cell type differences covariates included disease status and patients. Genes with at least a two-fold change and an FDR value ≤ 0.05 among comparison were considered significant and termed as differentially expressed genes (DEGs). Quantile normalized RPKM values were used in the heat maps and z-score values were plotted for genes visualization.

### Cytospin of sorted ASC populations

Cytospin was performed as previously described by our lab^1^. FACS-sorted ASC populations from PBMC were pelleted at 1300 rpm for 5 minutes on the Cytospin 4 (Thermo Scientific, Waltham, MA). Approximately 5,000 cells per population were dried overnight on albumin-coated slides and stained with Wright-Giemsa stain.

### Transmission Electron Microscopy

Transmission Electron Microscopy (TEM) was performed as previously described by our lab^10^. Briefly, FACS-sorted ASC populations were pelleted by centrifugation at 500g for 5 minutes. Pellets were then resuspended in PBS with 1 × 10^6^ erythrocytes to help visualize the pellets during the TEM processing. The pellets were then fixed (overnight, at 4° C) using 2.5M glutaraldehyde and then placed in 0.1% osmium tetroxide in 0.1M phosphate buffer (pH 7.4) for 1 h. Dehydration of pellets was performed by sequential incubation in ethanol solutions at different concentrations (25%, 50%, 75%, 95% and 100% EtOH). The pellets were then infiltrated, embedded, and polymerized using Eponate 12 resin (Ted Pella Inc.). Leica Ultracut S ultramicrotome was used to cut sections ~70 nm thick, which were then stained with 5% uranyl acetate and 2% lead citrate. JEOL JEM-1400 TEM (JEOL Ltd) with Gatan US1000 2k × 2k CCD camera (Gatan) were used for imaging.

### Statistical Analysis

Graphpad Prism version 8 software was used for statistical analysis. Statistical comparisons between groups were performed using one-way ANOVA, or Mann Whitney U test. The Welch’s t test was used to compare unmatched samples. The Holm-Sidak correction was used for multiple comparison analyses. Adjusted p-values were indicated as * p < 0.05, ** p < 0.01, *** p < 0.001, ****p < 0.0001. All error bars showed the mean ± standard deviation.

### Data availability

The AIRR sequencing and RNA sequencing datasets for ASC populations were deposited at Genome Expression Omnibus under the accession number GSE235660 and reviewer token spohswuazlwnhez.

### Code availability

Custom scripts and in-house developed analysis software were used with R, Matlab or Circos tools for visualization AIRR sequencing data, and the scripts are available at https://github.com/chenwr56/airr.

## Results

### Complexity and magnitude of circulating antibody-secreting cells in active SLE

**Fig. 1.**
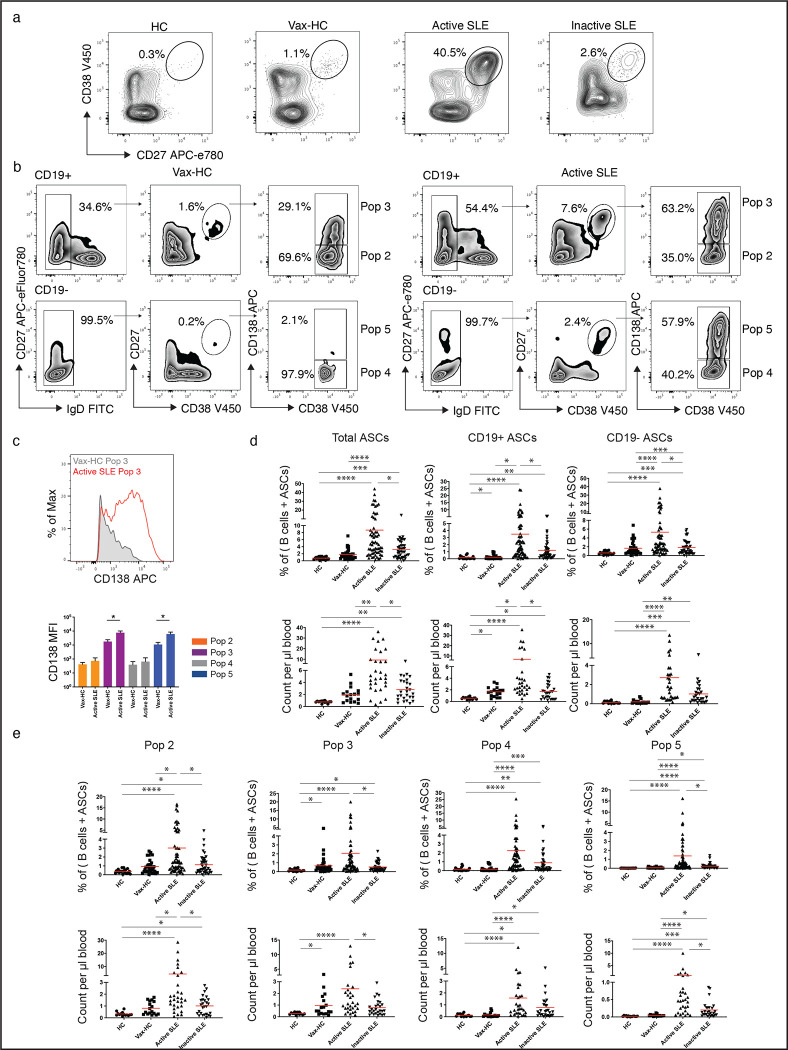
Complexity and Magnitude of Circulating Antibody-Secreting Cells in active SLE. Peripheral blood mononuclear cells (PBMCs) were isolated from steady-state healthy donors (n = 19), influenza vaccinated heathy subjects on day 7 post immunization (n = 33), active SLE patients (n = 63), or inactive SLE patients (n = 41). Isolated cells were stained and analyzed by flow cytometry. **a,** Representatives plots of the frequencies of CD19^+^ ASC (CD27^++^ CD38^++^) in the CD3^−^ CD14^−^ CD19^+^ gate. **b,** Gating strategy for the identification of ASC populations Pops 2–5, based on the CD19^+^ and CD19^−^ gate, in active SLE patients (left) and heathy subjects on day 7 post influenza immunization (right). **c,** Expression of surface CD138 on ASC populations were analyzed and shown as both representative histogram (top) and compiled data (bottom). **d,** Frequencies of total ASC, CD19^+^ ASC, and CD19^−^ ASC in total B cells and ASC combined (top), and numbers per μL of the peripheral blood (bottom). **e,** Frequencies of each ASC Pops 2–5, in the total B cells and ASC combined (top), and numbers per μL of the peripheral blood (bottom). (*p < 0.05, **p < 0.01, ***p < 0.001, ****p < 0.0001)

**Fig. 2.**
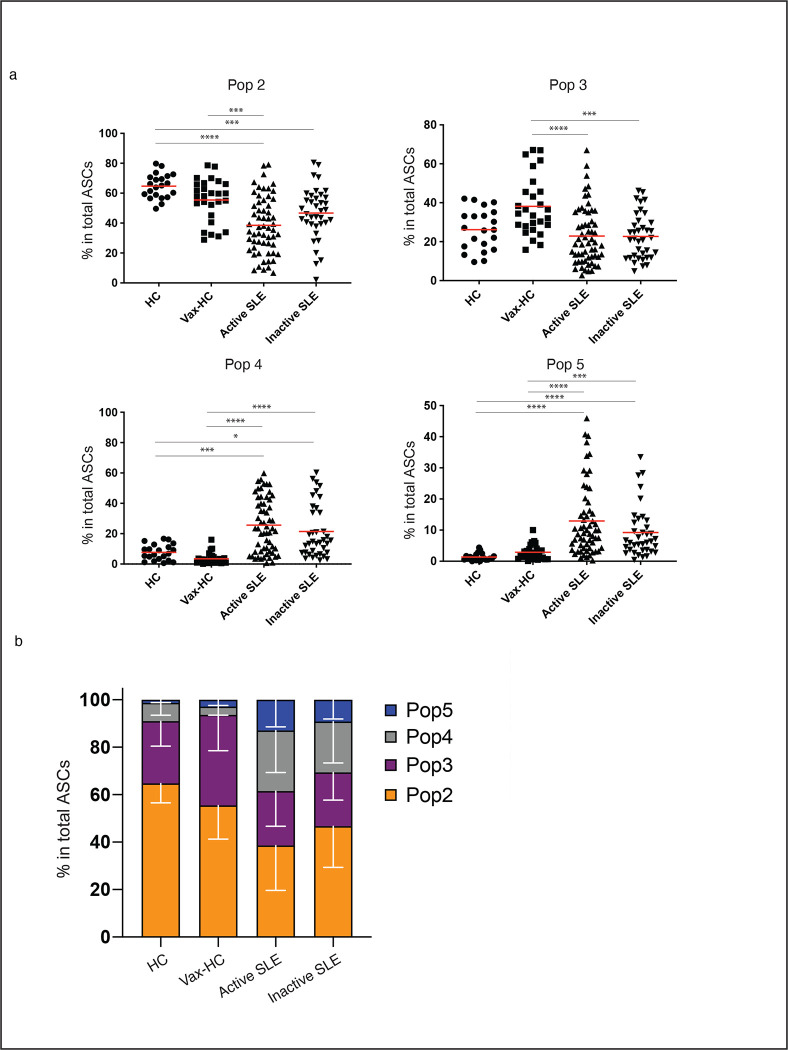
CD19^−^ ASC Substantially Contribute to the ASC Pool in Active SLE. **a,** Composition of each ASC population in total ASC in steady-state healthy donors (n = 19), influenza vaccinated heathy subjects on day 7 post immunization (n = 33), active SLE patients (n = 63), or inactive SLE patients (n = 41). (*p < 0.05, ***p < 0.001, ****p < 0.0001). **b,** Stacked bars showing the distribution of each Pop of ASC (Pop 2, Pop 3, Pop 4 and Pop 5) for the indicated groups. Data are shown as grouped analysis with S.D.

We employed the flow cytometry to characterize the abundance and diversity of circulating ASC in SLE, relative to the newly-generated ASC in the circulation of healthy subjects after recall immunizations. As depicted in Fig. 1a, SLE patients exhibited a greatly increased abundance of ASC relative to vaccinated healthy controls (vax-HC) and patients with inactive SLE. These results align with previous studies, including our own research^3, 6^. Notably, this study employed simultaneous evaluation of multiple phenotypic markers, yielding novel insights into the heterogeneity of expanded ASC, especially the more mature CD19^−^ compartment, which had remained understudied in SLE. Building upon our previous investigation of PC in healthy control (HC)^1, 11^, we identified four distinct populations of IgD^−^ CD27^++^ CD38^++^ ASC based on the expression of CD19 and CD138: Pop 2 (CD19^+^ CD138^−^), Pop 3 (CD19^+^ CD138^+^), Pop 4 (CD19^−^ CD138^−^), and Pop 5 (CD19^−^ CD138^+^) (Fig. 1b). Importantly, the levels of CD138 expression were significantly higher in CD138^+^ ASC populations in SLE relative to their counterparts in Vax-HC (Fig. 1b, c). We had described an earlier pre-PB population (Pop 1: CD19^+^ IgD^−^ CD27^−^ CD38^+^)^11, 12^, which is distinguished from transitional and pre-germinal center (GC) cells by the absence of CD10 and CD24, markers that were not included in the current analysis^12^, and Pop 1 was thus not quantified here. Overall, both the total ASC and CD19^+^ and CD19^−^ ASC fractions were increased in terms of relative frequencies and absolute numbers in both SLE groups relative to steady-state HC. Active SLE also displayed higher numbers of both ASC fractions relative to vax-HC and inactive SLE. However, only CD19^−^ ASC showed a significant elevation in inactive SLE relative to vax-HC (Fig. 1d). Among the four ASC populations, the most pronounced expansions were observed for CD19^−^ Pop 4/5 between active SLE and vax-HC, although significant differences were also detected for CD19^+^ Pop2/3 between these two groups (Fig. 1e, 2). Furthermore, significant differences were present between active and inactive SLE, except for Pop 4 (Fig. 1e), an ASC population that, as illustrated by the expression of various markers, appears to be heterogeneous in composition and may include fractions outside the ASC lineage. Overall, the contribution of CD19^−^ Pop 4/5 was greatly increased in both SLE groups relative to vax-HC, where they are scarcely detected, consistent with previous studies^13, 14^ (Fig. 2). Noteworthy, the abundance of both CD19^+^ and CD19^−^ ASC fractions, as well as individual ASC populations, correlated significantly with SLE disease activity, with the strongest correlations observed for CD19^+^ ASC and Pops 2, 3 and 5 (Supplemental Fig. 1).

### Immune phenotype of circulating ASC in active SLE

**Fig. 3.**
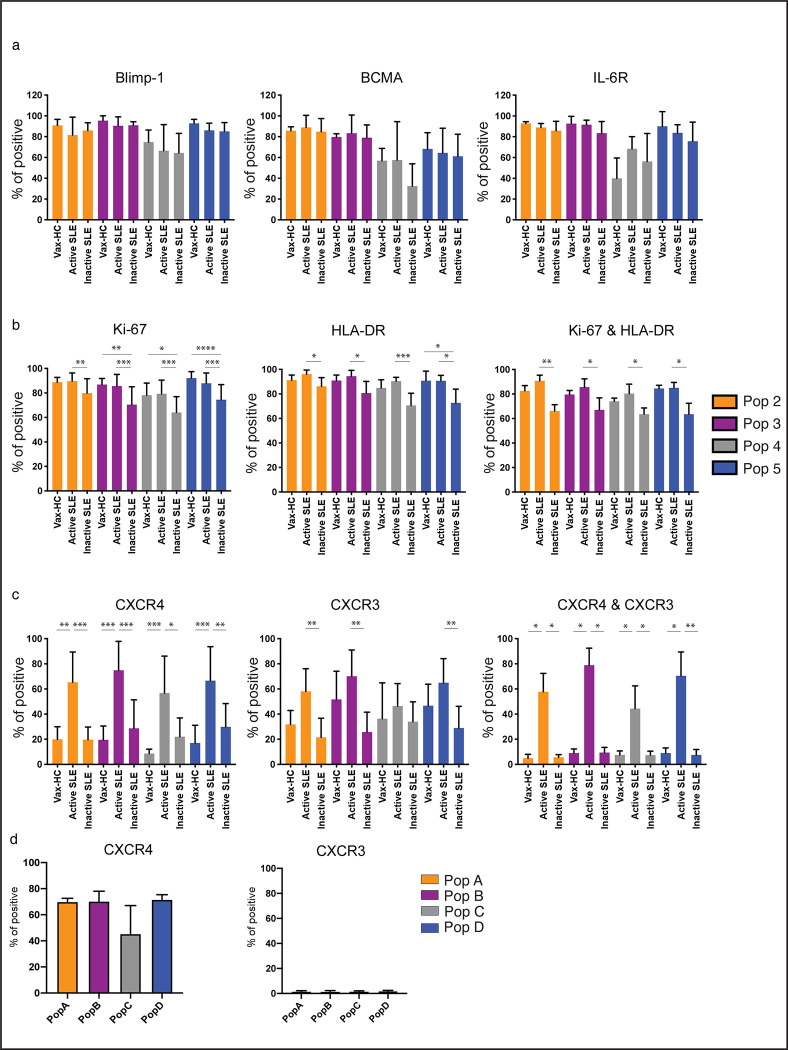
Immune Phenotype of Circulating ASC in Active SLE. **a-c,** Peripheral blood mononuclear cells (PBMCs) were isolated from steady-state healthy donors, influenza vaccinated heathy subjects on day 7 post immunization, active SLE patients, or inactive SLE patients, isolated cells were stained and analyzed by flow cytometry. **a,** Expression of intracellular molecular Blimp-1, and surface molecules BCMA and IL-6R on ASC populations (n = 8). **b,** Expression of intracellular Ki-67, surface HLA-DR and their co-expression on ASC populations (n = 15). **c,** Expression of surface expression of CXCR3, CXCR4, and their co-expression on ASC populations (n = 20). **d,** BM mononuclear cells (BMMCs) were isolated from SLE patients, and isolated cells were stained and analyzed by flow cytometry for the expression of CXCR4 (left) and CXCR3 (right) on BM PC populations, Pop A-D, from SLE patients. (n = 5). All pairwise comparison including disease status and ASC populations was analyzed, and the statistical significance showed here is only the comparison among disease groups within the same population. (*p < 0.05, **p < 0.01, ***p < 0.001, ****p < 0.0001)

To better understand the nature of circulating SLE ASC, we examined the expression of signature regulators of PC differentiation and survival including Blimp-1, BCMA and IL-6R (Fig. 3a). Blimp-1, a TF driving the differentiation of B cells into PC^15, 16^, was expressed at consistently high levels in all ASC populations but Pop 4, a notable fraction of which lacked Blimp-1 expression. BCMA and IL-6R, which play crucial roles in ASC survival^17, 18^, exhibited a similar pattern of expression, further confirming heterogeneity within Pop 4.

ASC can be categorized as proliferative, immature PB, or quiescent, mature PC, with the former population enriched in active peripheral immune responses, and the latter predominantly found in the stable, pre-formed BM PC compartment^1^. Majority of SLE ASC demonstrated ongoing or recent proliferation, as indicated by Ki-67 expression at levels comparable to PB in vax-HC. Of note, a predominance of Ki-67^+^ cells were present in all SLE ASC populations, both in active and inactive disease, although inactive SLE exhibited a significantly larger fraction of Ki-67^−^ cells in all ASC populations (Fig. 3b). Consistent with other studies^3^, Ki-67 was tightly associated with the HLA-DR expression, a marker that is downregulated during PC maturation. Interestingly, uncoupling of Ki-67 and HLA-DR was observed in a substantial fraction of inactive SLE ASC, suggesting a more heterogeneous compartment in these patients (Fig. 3b, Supplemental Fig. 2a). Overall, active SLE and vax-HC ASC displayed a relatively homogenous early proliferative/HLA-DR^+^ phenotype, even in cells with an otherwise mature BM-like phenotype, i.e., CD19^−^CD138^+^ fraction (Pop 5).

The ability of ASC to migrate to non-lymphoid tissues or the BM is determined by the expression of CXCR3 and CXCR4, chemokine receptors that promotes migration to inflammatory tissues induced by Th1 responses and to the BM, respectively^19, 20, 21, 22^. CXCR4 was expressed in the majority of circulating SLE ASC at significantly higher levels than vax-HC and inactive SLE (Fig. 3c, Supplemental Fig. 2b). In contrast, CXCR3 was overexpressed in active SLE compared to inactive SLE, with a similar but not statistically significant trend observed relative to vax-HC. Notably, the majority of ASC in active SLE co-expressed CXCR3 and CXCR4, whereas dual expression of these receptors was present at very low levels (<10%) in both inactive SLE and vax-HC. Supporting the role of CXCR4 in ASC homing and retention in the BM, even in the pro-inflammatory milieu of the lupus BM, this receptor was expressed in the majority of BM PC whereas CXCR3 expression was rare on SLE BM PC (Fig. 3d).

Previous studies have reported that in steady-state HC, the majority of circulating ASC are IgA-producing PB, with a surge of antigen-specific IgG ASC observed 6–7 days after systemic immunization^23, 24^. In the context of SLE, it has been suggested that IgA ASC may constitute a significant portion of ASC (up to 90%; 58% on average)^25^. However, it is important to note these studies were conducted on European patients with low disease activity. In our predominantly African American cohort, patients with active SLE exhibited a dominance of circulating IgG ASC over IgA ASC, with the highest ratio observed within Pop 5. Conversely, IgG/IgA ASC ratios were consistently lower in inactive SLE patients. Furthermore, IgG/IgM ASC ratios were significantly increased in active SLE compared to inactive SLE (Supplemental Fig. 3a, b). Notably, in inactive SLE, IgA ASC were increased in the non-proliferative (Ki-67^−^) relative to proliferative fraction (Ki-67^+^) (Supplemental Fig. 3c). Overall, ASC expansions in active SLE were primarily composed of IgG-producing cells, while IgA ASC predominated in inactive SLE, replicating the characteristic profile of acute immune responses and steady-state condition, respectively.

### Proliferative peripheral SLE ASC display enhanced mature morphology

**Fig. 4.**
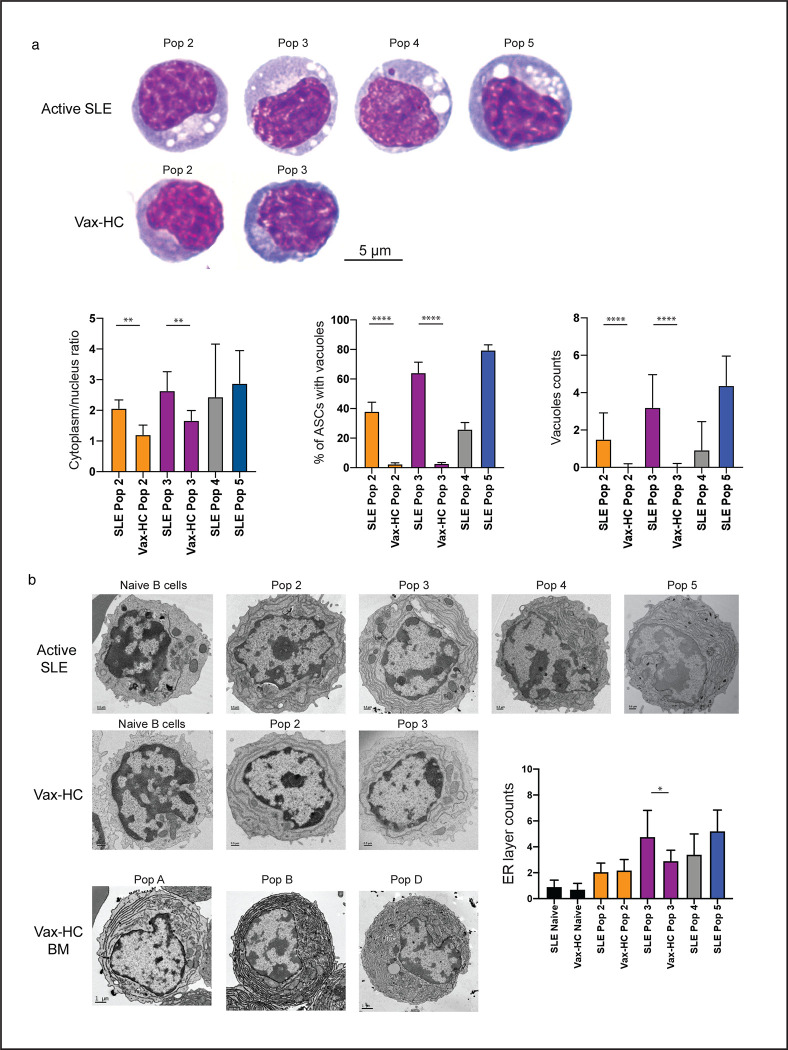
Proliferative Peripheral SLE ASC Display Enhanced Mature Morphology. **a,** Wright-Giemsa staining of FACS sorted peripheral ASC populations from active SLE patients and influenza vaccinated heathy subjects on day 7 post immunization. Representative images (top) and compiled data for quantification of cytoplasm/nucleus ratio and vacuoles of the 100X magnification of ASC. **b,** Electron microscopy of ASC populations sorted from influenza vaccinated heathy subjects and active SLE patients. Representative images and compiled data show the changes of ER layers in ASC from active SLE. Mature bone marrow PCs from vaccinated heathy subjects were also included for reference. Statistical difference is shown among disease groups within the same population. (*p < 0.05, **p < 0.01, ****p < 0.0001)

One remarkable finding from our studies was the significant expansion of CD19^−^ ASC, particularly the CD19^−^CD138^+^ fraction (Pop 5), which displayed a surface phenotype resembling more mature BM PC^1^. Despite this resemblance, the large majority of ASC in active SLE, including Pop 5, demonstrated features of recent generation and active or recent proliferation, as indicated by the coordinated expression of HLA-DR and Ki-67. Morphologically, these ASC in active SLE exhibited characteristic consistent with a mature phenotype. Similar to mature PC in the healthy BM, all ASC populations in SLE showed a higher cytoplasm/nucleus ratio and prominently displayed an increased number of cytoplasmic vacuoles, which are typical of mature BM PC^1^ (Fig. 4a). Electron Microscopy (EM) studies further confirmed the presence of vacuolar structures and revealed an expanded endoplasmic reticulum (ER) structure in ASC from active SLE, particularly in Pop 3 from SLE compared to vax-HC. Interestingly, peripheral blood Pop 5 from SLE patients exhibited ER complexity similar to the most mature population (pop D) observed in healthy BM (Fig. 4b).

### Heterogeneous SLE ASC responses share common precursors

**Fig. 5.**
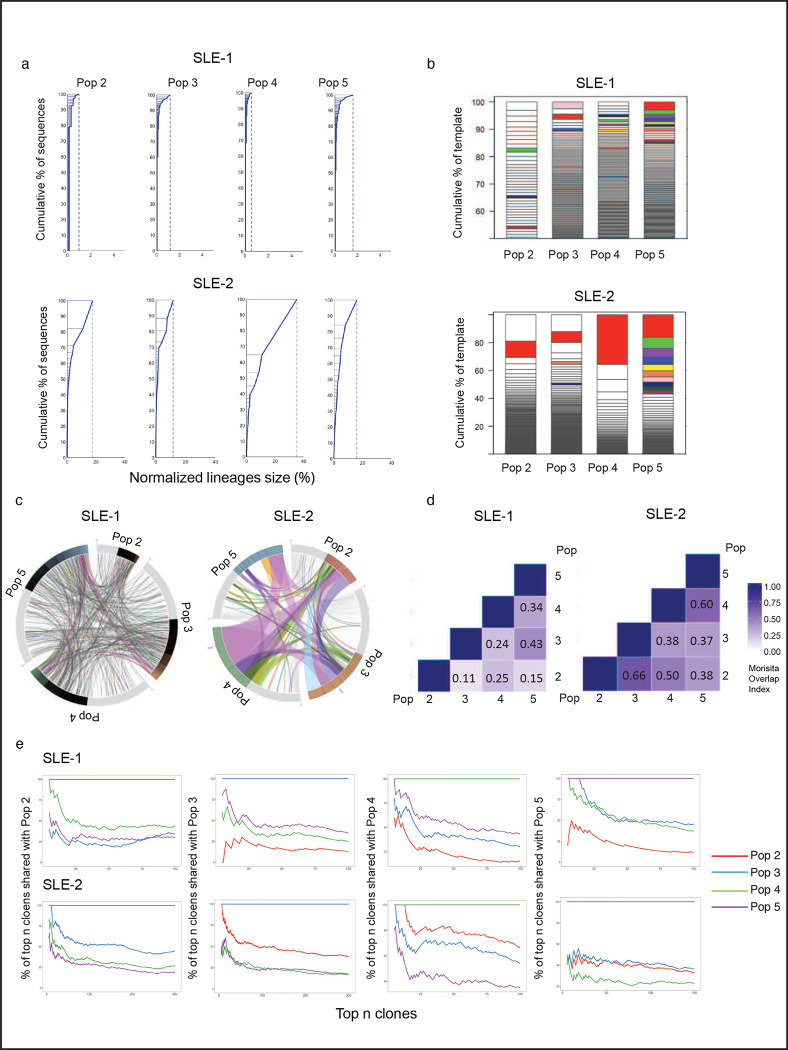
Heterogeneous SLE ASC Responses Share Common Precursors. AIRR-seq was used to analyze the clonal repertoire of ASC populations from 2 active SLE patients. **a,** Clonality of the repertoire in ASC populations is shown by plotting normalized lineage size versus the cumulative percent of sequences. Lineages are size-ranked in descending order along the extent of the y-axis representing 100% of all the sequences. Horizontal lines delineate the individual lineages. **b,** Stacked bar plots demonstrate the diversity of the repertoire by showing descending, size-ranked clones as segments comprising percentages of the total repertoire. The largest 10 clones of the reference population pop 5 are colored, and like-colors in other populations show identical clones in other populations. **c,** Circos plot shows interconnectedness of the ASC populations by plotting the sequences from each population in clonal size-ranked order with the largest clones being the most clockwise portion of each population segment. Lines between ASC populations indicate matched clones between ASC populations. **d,** The Morisita Overlap Index demonstrates the similarity of repertoires in various ASC populations as a value from 0 (no similarity) to 1 (identical repertoire). The color strength is indicative of interconnectivity. **e,** The clonal relatedness of SLE ASC populations is shown by plotting the percent of shared clones with Pop 2, Pop 3, Pop 4, and Pop 5, respectively (y axis), within the top numbers of clones (x axis).

The question of whether different types of ASC, including immature PB and mature PC, arise from distinct B cell precursors is still not fully understood. Also unknown is whether a common precursor might generate different ASC progeny through separate differentiation pathways, or instead through sequential maturation. To address this question, we performed next generation sequencing of the antibody repertoire expressed by ASC populations in active SLE (Supplemental Table 1). The results of VDJ sequencing confirmed our previous findings, showing ASC in most SLE patients exhibited a predominantly polyclonal repertoire^6^. However, in some individual patients, the ASC repertoire was dominated by large clonal expansions that were detectable in all populations, providing an informative window into their cellular origin (Fig. 5a and Supplemental Fig. 4). Shared clones were observed among ASC populations (Fig. 5b, c) and quantified through the Morisita index (Fig. 5d) in both types of patients. A large proportion of ASC clones detected in the CD19^−^ Pops 4/5 were present in the CD19^+^ Pops 2/3, and likewise, substantial connectivity were observed in ASC population with or without CD138 expression (Fig. 5d). Notably, ASC clones identified as abnormally expanded, defined by a difference threshold of 0.1% larger than the previous clone in size-ranked clones^6^, displayed higher degrees of inter-population connectivity (Supplemental Fig. 5a). Even within the same-day blood samples, which inherently underestimate connectivity between cellular compartments due to unsynchronized development and/or different clonal persistence, the more mature CD19^−^ CD138^+^ (Pop 5) displayed up to 14% global connectivity with the more immature CD19^+^ CD138^−^ (Pop 2) among expanded clones. Among the 25 largest clones present in Pop 5, 35% to 40% of clones were also documented in Pop 2 (Fig. 5e). Overall, the load of somatic hypermutation (SHM) was similar across all populations. Despite the high clonal connectivity between ASC populations, no identifiable intraclonal sequence divergence or sequential progression of somatic mutation was observed. These findings supports a model of longitudinal maturation acting upon a common post-GC precursor (Supplemental Fig. 5b).

### SLE ASC express a distinct transcriptome

**Fig. 6.**
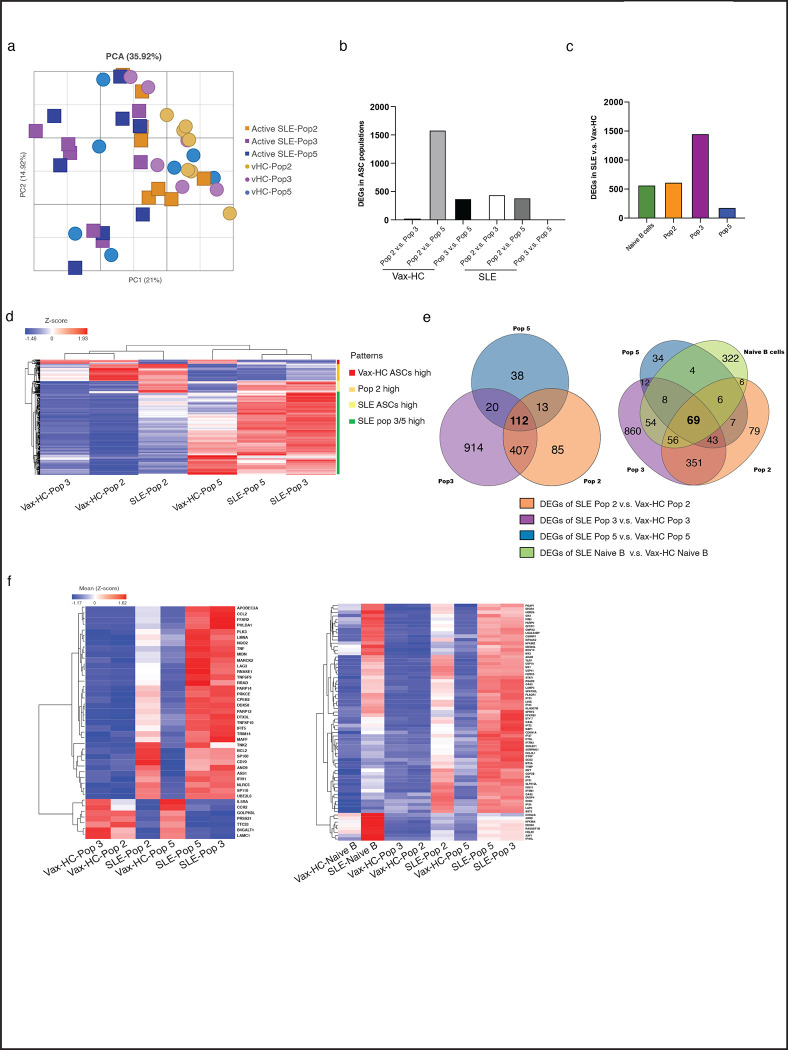
SLE ASC Express a Distinct Transcriptome. Peripheral ASC populations from 9 active SLE patients and 7 healthy subjects post influenza vaccination were sorted and prepared for bulk RNA sequencing to compare their transcriptome. **a,** Principal component analysis (PCA) of differentially expressed genes (DEGs) (at least two-fold change of expression and FDR < 0.05) detected from all pairwise comparisons of the six groups. **b**, Bar plots show DEGs within populations from same disease groups, including both active SLE patients and heathy subjects post vaccination, respectively. **c,** Bar plots show DEGs between SLE and post-vax HC for each ASC population and naïve B cells. **d,** Heatmap of z-score normalized RPKM expression for all DEGs identified, and four differentiating patterns were identified and indicated in the legend. Data represent the mean expression for each group. **e,** Venn diagrams show the overlap of DEGs among all ASC Pops identified from the comparison of SLE and vaccinated HC (left), and overlap of DEGs among all ASC Pops and naïve B cells by comparing SLE and vaccinated HC (right). **f,** Heatmap of z-score normalized RPKM expression for the overlapped 43 DEGs that are shared in all ASC populations but not in naïve B cells, and for the 69 genes that are shared in all ASC populations and naïve B cells from the Fig. 6e.

We have previously reported the transcriptional and epigenetic programs of peripheral and BM ASC in HC^10, 11, 26, 27^. Here, we performed RNA-seq of ASC Populations to gain insights into disease-specific developmental and survival programs that may contribute to the observed phenotypic and morphological differences between SLE and HC ASC. A substantial number of Differentially Expressed Genes (DEGs) were identified between the corresponding populations in active SLE relative to vax-HC (Fig. 6). In general, SLE and HC ASC were primarily separated by a first principal component, which contributed 21% of the total DEGs identified (Fig. 6a). Consistent with our previous findings in HC^10, 13^, the physiological ASC transcriptome suggested a transition from highly similar Pops 2/3 to Pop 5, with a gradual extinction of certain transcripts and acquisition of a larger set of DEGs, resulting in a significant number of DEGs between HC Pop 2 and 5 (Fig. 6b, d). In contrast, reflecting enhanced maturation based on phenotypic and morphological features, the most significant transcriptional difference in SLE ASC was observed between Pop 2 and Pop 3, which in turn showed high similarity to Pop 5 (Fig. 6b, d). Regarding the comparison among SLE and vax-HC, the largest difference was detected in Pop 3, with the majority of DEGs representing over-expressed genes in SLE (Fig. 6c, d). More than 100 DEGs were found to be shared across all SLE ASC populations relative to vax-HC (Fig. 6e), including interferon (IFN)-dependent genes and a collection of highly immunologically relevant genes that are not IFN-dependent (Fig. 6f). Notably, among these overlapping DEGs, a subset of 69 genes were initially differentially expressed in SLE naïve B cells(Fig. 6e, f). The presence of the shared DEGs aligns with our previous recognition of DEGs overexpressed in SLE naïve B cells relative to HC, with propagation of this pattern across activated effector and memory B cells, a feature might be determined by epigenetic regulation^9, 28^.

Gene set enrichment analysis (GSEA) identified signaling pathways that exhibited consistent hyperactivity across all SLE ASC populations, predominantly type I and Type II IFN. The generic SLE ASC pattern also indicated increased activity in TNF-α signaling, inflammatory responses, estrogen responses, and IL-6 signaling. These SLE-associated pathways were particularly prominent in Pops 3/5, which were also significantly enriched for IL-2/STAT5 signaling, and anti-apoptotic programs, as further discussed below. On the other hand, the generic vax-HC ASC profile was characterized by enhanced glycolysis, fatty acid metabolic programs, UV response and TGF-β signaling (Supplemental Fig. 6a). In SLE, TNF-α and other inflammatory pathways were predominantly pronounced in SLE Pops 2/3 relative to vax-HC, and more attenuated, albeit still over-expressed in the more mature SLE Pop 5 (Supplemental Fig. 6b). KEGG analysis corroborated the findings from GSEA pathways, and it also revealed the over-expression of additional innate immune pathways, including TLR^29, 30^, NOD^31, 32^ and RIG-I^33^, all of which have significance in SLE and other autoimmune diseases, and have been previously observed to be upregulated in B cells from SLE in our lab ^6, 8, 9^ (Supplemental Fig. 6c).

**Fig. 7.**
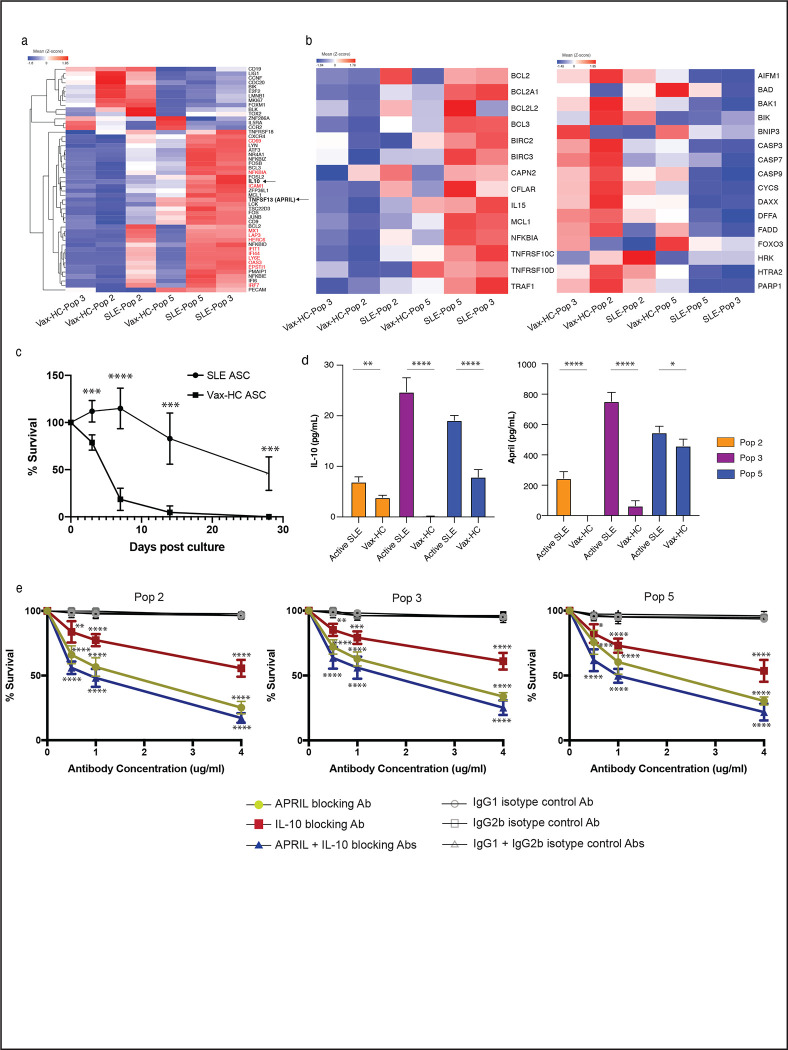
SLE ASC Display Enhanced In Vitro Survival. **a,** Heatmap of z-score normalized RPKM expression for select genes are shown with IFN-stimulated genes colored in red. Data represent the mean expression for each group. **b,** Heatmap of z-score normalized RPKM expression for anti-apoptotic genes (left) and pro-apoptotic genes (right) in ASC Pops from SLE and healthy subject post vaccination. Data represent the mean expression for each group. **c,** Sorted CD19^+^ ASC from SLE and post-vax HC were cultured in MSC media and their survival in vitro was measured on day 0, 3, 7, 14 and 28 post culture by IgG ELISpot and normalized to the IgG ELISpot numbers of sorted ASC prior to culture. **d,** Sorted ASC Pops 2, 3, and 5 from active SLE and post-vax HC were cultured in MSC media, and the supernatant harvested on day 3 to detect the secretion of IL-10 and APRIL by ELISA. The statistical significance showed here is the comparison among disease groups within the same ASC Pops. **e,** Sorted ASC Pops 2, 3, 5 from active SLE were cultured in MSC media with neutralizing antibody against IL-10, APRIL, or both, or their corresponding isotype controls at concentrations of 0.5 ug/ml, 1 ug/ml and 4 ug/ml, and cell survival was measured by IgG ELISpot on day 3, normalized to ASC cultured in the absence of antibodies, and compared with corresponding isotype controls. (*p < 0.05, **p < 0.01, ***p < 0.001, ****p < 0.0001)

Notably, in SLE ASC Pops 3 and 5, there was a significant increase in the expression of genes associated with ASC differentiation and/or survival. These included IL-10^34, 35^, APRIL (TNFSF13)^17, 36, 37^, as well as anti-apoptotic genes, adhesion and homing markers (CD31/PECAM, CD54/ICAM1, CD69, and CXCR4), and BCR signaling components including NR4A1 (Nur77)^38^ and Lyn^39, 40^, JUNB and FOS, and ATF3 (Fig. 6a). Moreover, SLE ASC exhibited significantly higher expression of anti-apoptotic genes including BCL2 and MCL1, and concurrent downregulation of pro-apoptotic genes including caspases, BAD, BAX, BIK and FADD^41^. Notably, BCL2 expression was most pronounced in SLE Pop 2 and remained at high levels in SLE Pops 3/5. In contrast, high expression of MCL1 was acquired by SLE Pops 3/5. Additionally, these two populations showed high expression of TNF receptor superfamily member 10c and 10d, which serve as decoy receptors and inhibit TRAIL-induced apoptosis^42^ (Fig. 7b).

### SLE ASC display enhanced in vitro survival

To validate the transcriptomic disparities in survival programs, we sorted ASC from the blood of active SLE patients and vax-HC to assess their survival ability in our in vitro model using mesenchymal stomal cells secretome. Compared to vax-HC ASC, SLE ASC displayed higherviability even within the initial days of culture, and by day 28, half of the SLE ASC remained viable, whereas nearly all ASC from vax-HC perished (Fig. 7c). Subsequent culture studies provided further evidence for the enhanced secretion of IL-10 and APRIL by SLE ASC, in line with the corresponding transcriptional levels (Fig. 7d). Crucially, the survival of SLE ASC was significantly compromised when cultured in the presence of blocking antibodies against IL-10, APRIL, or both. In contrast, cultures treated with antibody isotype controls exhibited no notable impact on ASC viability. These findings underscore the critical role of these autocrine loops in sustaining the survival of SLE ASC (Fig. 7e).

## Discussion

Given the importance of autoantibodies in SLE, it is critical to understand their mechanistic basis. The overall abundance of peripheral ASC (PB) correlates with disease activity as evidenced by phenotypic and modular transcriptional analyses. It has also been postulated that autoreactive ASC (PC) may be increased in the SLE BM, since high titers of certain autoantibodies persist even after B cell depletion, suggesting sustained production by autonomous LLPC. These features could be explained by increased PC generation during active disease, accompanied by enhanced maturation and homing to the BM, as well as prolonged survival mediated by intrinsic programming and/or extrinsic cues. However, these questions have not been fully addressed due to the limitations of existing studies, which have employed unfractionated samples containing various ASC (including PB and PC), utilized limited surface markers, and lacked comprehensive RNA sequencing^43^.

In order to address these knowledge gaps, we present the first in-depth characterization of the multiple circulating ASC populations present in the blood of SLE patients, comparing them to ASC that typically surge 7 days after recall immunization in HC but subsequently regress to normal levels within 2 weeks, a profile that has been ascribed to programmed apoptosis that regulates the duration of the acute immune response. It has been proposed that circulating ASC may represent, at least in part, the displacement of pre-existing BM PC by new arrivals competing for survival niches during recall responses ^44^.

Our findings confirm the major increase in the global amount of circulating ASC in active SLE, while also provide the first evidence of a substantial expansion of CD138^+^ cells lacking CD19 expression, a phenotype typically ascribed to mature BM LLPC^1, 14^. The increased generation of CD19^−^ ASC has important implications for the SLE treatment strategies involving anti-CD19 agents, including monoclonal antibodies^45^ and CD19 CART cells^46, 47^. However, in contrast to terminally differentiated resting BM PC, even the most differentiated circulating SLE ASC display a proliferative/HLA-DR^+^ phenotype akin to immature ASC observed in early immunization responses. This profile argues against BM displacement as a major contributor to the expansion of peripheral ASC in active SLE. Notably, in inactive SLE, a significant fraction of ASC lacked expression of Ki-67 and/or HLA-DR, with an average of 40% of all ASC populations lacking both markers. This finding could be explained either by equal displacement of all BM PC populations, or by the chronic generation and prolonged peripheral persistence of newly produced ASC in inactive SLE. The latter scenario could also account for the prevalence of IgA ASC in inactive disease, similar to steady-state HC^24, 25^. This contrasts with the IgG dominance observed in active SLE and in HC following systemic immunization, thereby suggesting distinct sources of ASC in different SLE states, with a predominance of low-grade proliferation of housekeeping mucosal IgA in inactive disease. Further investigation is needed to elucidate the extended phenotype of homing markers, antibody repertoire, and antigen-specificity to confirm this possibility.

Regardless, VDJ repertoire sequencing clearly demonstrates a large degree of clonal sharing across all ASC populations in active SLE. Together with the lack of intraclonal accumulation of SHM, these findings demonstrate the existence of shared B cell precursors with the potential to generate ASC at different stages of maturation and favor a model of longitudinal maturation acting upon a common post-GC precursor.

SLE ASC display several phenotypic features that provide important insights into their abnormal functions, which might underlie the multiple pathogenic aspects associated with the disease. In addition to their enhanced generation and higher numbers of mature CD19^−^ ASC, multiple properties contribute to their accumulation and survival in the BM. This process eventually leads to the large reservoir of autoantibody-producing PC, which explains the presence of stable autoantibodies characteristic of SLE, prominently including anti-RNA binding antibodies such as anti-Ro and anti-Smith/RNP. Notably, SLE ASC exhibit distinctive characteristics, including high levels of the chemokine receptors CXCR4 and CXCR3, often in combination. CXCR4 plays a central role in the homing of PC to the BM and their retention in long-lived niches. It may also contribute to the migration and retention of ASC in Lupus kidneys^20, 48^, an activity also promoted by CXCR3 induced by Th1-like responses^22^. Therefore, the enhanced co-expression of CXCR4 and CXCR3 in circulating SLE ASC suggests a strong capability of these cells to migrate into inflamed Lupus kidneys, whether as a primary or a secondary amplifying event. Of note, increased CXCR4 transcription was closely paralleled by the transcriptional levels of the RNA-binding protein ZFP36L, with maximal expression observed in SLE ASC Pop 3 and 5. ZFP36L deficiency is known to limit the abundance of molecules involved in ASC homing to the BM, and its absence leads to diminished accumulation of ASC in the BM^49^.

Other mechanisms contributing to increased generation and survival of SLE ASC included their enhanced expression of CD138 and the elevated production of IL-10 and APRIL. IL-10 is a potent mediator of B cell proliferation and PC differentiation and can be produced by various cell types in SLE, including peripheral Th10 CD4 T cells ^34, 35, 50^. Our data demonstrate that SLE ASC can also produce IL-10, creating an autocrine loop. IL-10 production has been associated with regulatory functions in PC,^51^, therefore it appears counterintuitive it is increased in SLE ASC. However, previous studies have also demonstrated increased B cell activity with IL-10^52^, potential therapeutic benefits of IL-10 inhibition in SLE^53, 54^; and pro-inflammatory effects of IL-10 in an IFN-dominated milieu^55^, suggesting a complex role for IL-10 in autoimmune diseases. Furthermore, our findings provide the first evidence of autocrine production of APRIL by ASC, which is substantially increased in SLE and enhances ASC survival in vitro. Acting through TACI and BCMA receptors, APRIL is an important regulator of plasma cell differentiation and survival^36, 56^, as also demonstrated in our previous work with a BM mimetic culture system^7^. Notably, CD138 was over-expressed in SLE ASC, and CD138-bound APRIL induces ASC differentiation independently of IFN, a mechanism that is enhanced in SLE^37^. Therefore, our results support the existence of an autocrine effect of APRIL production by SLE ASC, which may be further enhanced by the higher levels of CD138 expression observed in SLE. This autocrine loop could be of significant relevance to SLE pathogenesis and treatment, as highlighted by the therapeutic benefits of agents targeting components, such as TACI-Ig and anti-BCMA strategies, including BCMA CAR-T-cell therapy^57^.

Our study provides original insights into the transcriptional regulation of SLE ASC, demonstrating significant transcriptional changes in SLE ASC Pop 3. Overall, a distinct SLE transcriptome emerges, characterized by an inflammatory signature driven by type I and type II IFN, IL6, and TNF, and mediated by NF-κB signaling. A fraction of this signature was also shared by naïve B cells in SLE. These findings, combined with our previous SLE epigenetic studies, support the idea of an epigenetically determined transcriptional program propagated throughout B cell differentiation. Of particular interest are the shared over-expression of TRL7 and EPSTI1 (epithelial mesenchymal interacting protein 1) in SLE naïve B and ASC. TLR7 hyperactivity plays a major role in SLE development, including as monogenic B cell-intrinsic gain-of-function somatic mutations^58^, and in the activation of naïve and DN2 B cells, which leads to the generation of pathogenic ASC through extrafollicular pathway^8^. Additionally, we had shown that EPSTI1 is highly demethylated in SLE naïve B cells, and there is a strong correlation between demethylation and disease activity^9^. Interestingly, EPSTI1 is also overexpressed in Sjogren’s B cells and induces B cell activation and antibody production through NF-κB signaling^59^. SLE ASC, especially Pops 3/5, exhibit overexpression of AP-1 proteins JunB and ATF3. We have previously identified ATF3 as one of the top TF overexpressed in SLE B cells owing to enhanced chromatin accessibility ^9^. ATF3 facilitates the hetero-trimerization of AP-1 proteins, and JunB is essential for cell identity in other immune cells^60^. JunB also plays a central role in multiple myeloma cell proliferation, and drug resistance in the BM microenvironment^61^, and serves as a key regulator of myeloma BM angiogenesis^62^. Hence, these JunB-mediated mechanisms could contribute to the fate determination, accumulation, and resistance to treatment of SLE ASC. Finally, SLE ASC, particularly Pop 2, exhibit a strikingly high level of TOX2 expression. TOX2 is a TF that induces T follicular helper cells (Tfh) differentiation through enhanced BCL6 chromatin accessibility^63, 64^. TOX2 is also important for inducing and sustaining GC formation and promoting T-bet expression in B cells, which is most prominent in activated SLE naïve B cells and DN2 cells, both representing ASC precursors ^6, 8^.

In this study, we have also found that SLE ASC overexpress additional innate immune pathways, including TLR, NOD, and RIG-I, which were previously characterized as hyperactive in SLE DN2 cells, one of the main precursors of ASC in SLE^6, 8, 9^. Furthermore, TLR, NOD, TNF pathways, other pro-inflammatory cytokines, and BCR signaling could contribute to ASC stimulation through the canonical NF-κB signaling pathway^65^. On the other hand, the vax-HC ASC exhibit enriched glycolysis and fatty acid metabolic pathways. This observation may be consistent with a larger fraction of recently generated PB^66, 67, 68^. However, further studies are required to elucidate these critical cellular processes, which are beyond the scope of our current study.

Finally, our studies reveal that mature SLE CD138^+^ ASC (Pops 3/5), exhibit a transcriptional program favoring cell survival, characterized by increased expression of anti-apoptotic genes and concurrent downregulation of pro-apoptotic mediators. SLE ASC also display heightened levels of adhesion and homing receptors (PECAM1, ICAM1, CD69 and CXCR4), which facilitate migration and retention in protective survival niches. Specifically, PECAM1 and CD69 are likely to play crucial roles in the formation and migration of PC to the BM^69, 70 71, 72^, together with CXCR4 promoting the latter function^73^. ICAM1, preferentially expressed in mature BM PC, is essential for homotypic aggregation and survival^74^. Notably, SLE ASC show elevated expression of Nur77 (NR4A1), Lyn, and Jun, signaling molecules induced by BCR activation, which have been demonstrated in murine models to decrease PC survival and BM accumulation of PC^38, 40, 75^. Therefore, the overexpression of these molecules in SLE ASC may potentially represent an unsuccessful attempt to limit PC survival by antagonizing anti-apoptotic factors and counteracting cytokine responsiveness. Overall, this profile consistently reflects the generation and maturation of SLE ASC within a highly inflammatory environment, distinguishing it from conventional protein vaccines. This disparity may underlie many of the observed differences between SLE and vax-HC in our study. The inflammatory milieu likely amplifies the epigenetic abnormalities inherent in SLE B cells, thus providing a potent stimulus for ASC development.

In conclusion, this research offers a comprehensive understanding of the phenotypic, molecular, and functional aspects that clarify the mechanisms underlying the generation, survival, and behavior of SLE ASC. These findings will significantly contribute to the informed selection and application of available therapeutic agents capable of targeting specific ASC populations based on their expression of surface markers, such as CD19 and CD138, as well as their chemokine receptors, survival factors, and regulatory TF. Particularly noteworthy is the potential of utilizing bifunctional agents, including monoclonal antibodies, CAR-T cells or T cell engagers, to selectively target ASC populations responsible for active autoantibody production. By leveraging these insights, future interventions can be designed to effectively modulate and manage SLE pathogenesis.

## Supplementary Material

Supplement 1
