# Supplementary material for: SLE Antibody-Secreting Cells Are Characterized by Enhanced Peripheral Maturation and Survival Programs
Source: Res Sq. 2023 Jun 27:rs.3.rs-3016327. Preprint. [Version 1] doi: 10.21203/rs.3.rs-3016327/v1 (PMC10350208; doi:10.21203/rs.3.rs-3016327/v1)
Supplement: Supplement 1 [file NIHPPrs3016327v1-supplement-1.pdf]

Sup. Fig. 1

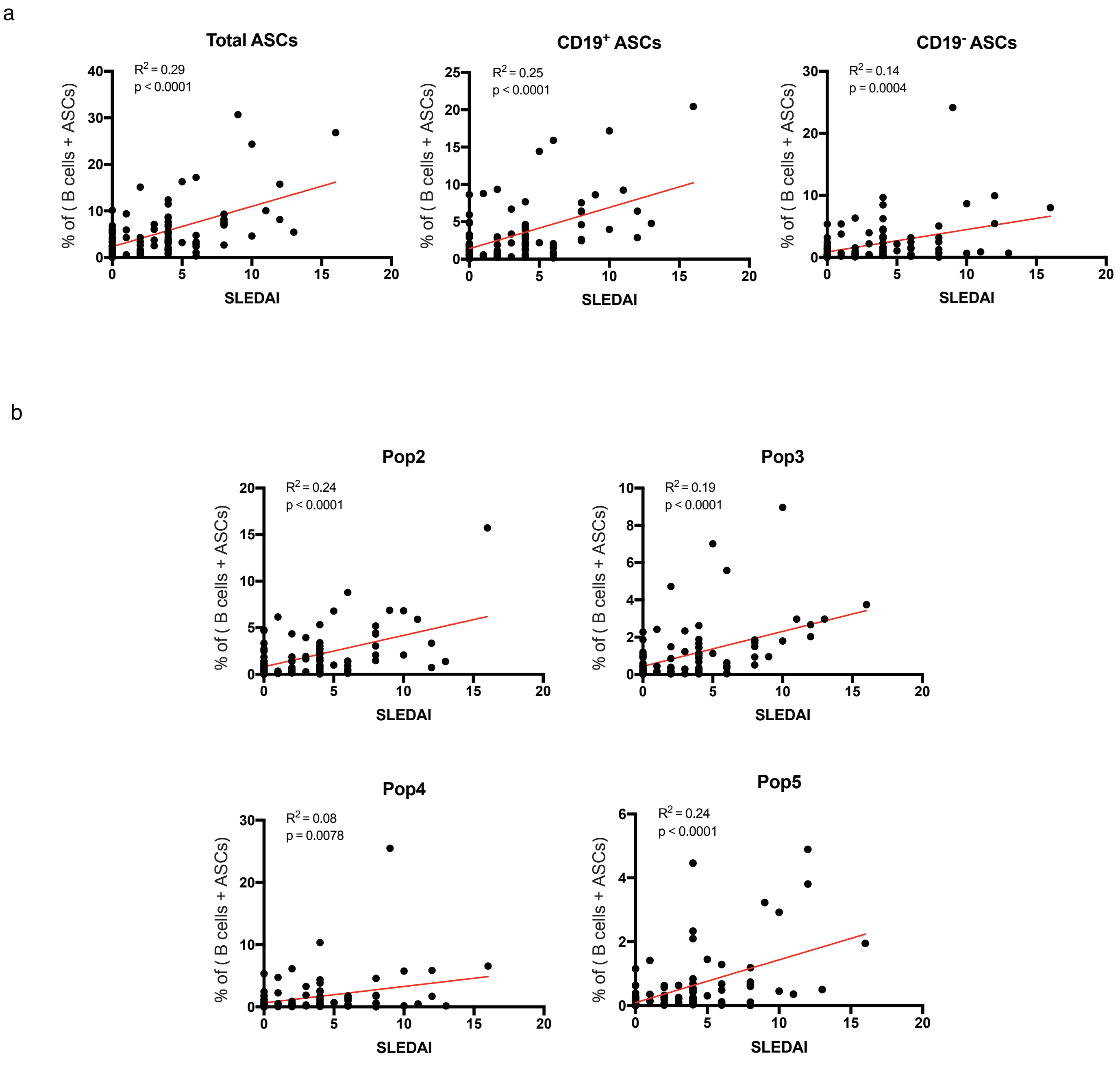

a
Total ASCs
R^2 = 0.29
p < 0.0001
CD19+ ASCs
R^2 = 0.25
p < 0.0001
CD19- ASCs
R^2 = 0.14
p = 0.0004
b
Pop2
R^2 = 0.24
p < 0.0001
Pop3
R^2 = 0.19
p < 0.0001
Pop4
R^2 = 0.08
p = 0.0078
Pop5
R^2 = 0.24
p < 0.0001
% of (B cells + ASCs)
SLEDAI

Supplemental Fig. 1. Circulating ASC and Populations are Correlated with SLE diseases Activity Index (SLEDAI).

a, Positive correlation between the disease activity and frequencies of total peripheral blood ASC, $CD19^+$ ASC, and $CD19^-$ ASC in total B cells and ASC combined using Spearman's r coefficient (n= 84). **b,** Positive correlation of disease activity and the frequencies of each ASC population in the peripheral blood in total B cells and ASC combined using Spearman's r coefficient (n= 84).

Sup. Fig. 2

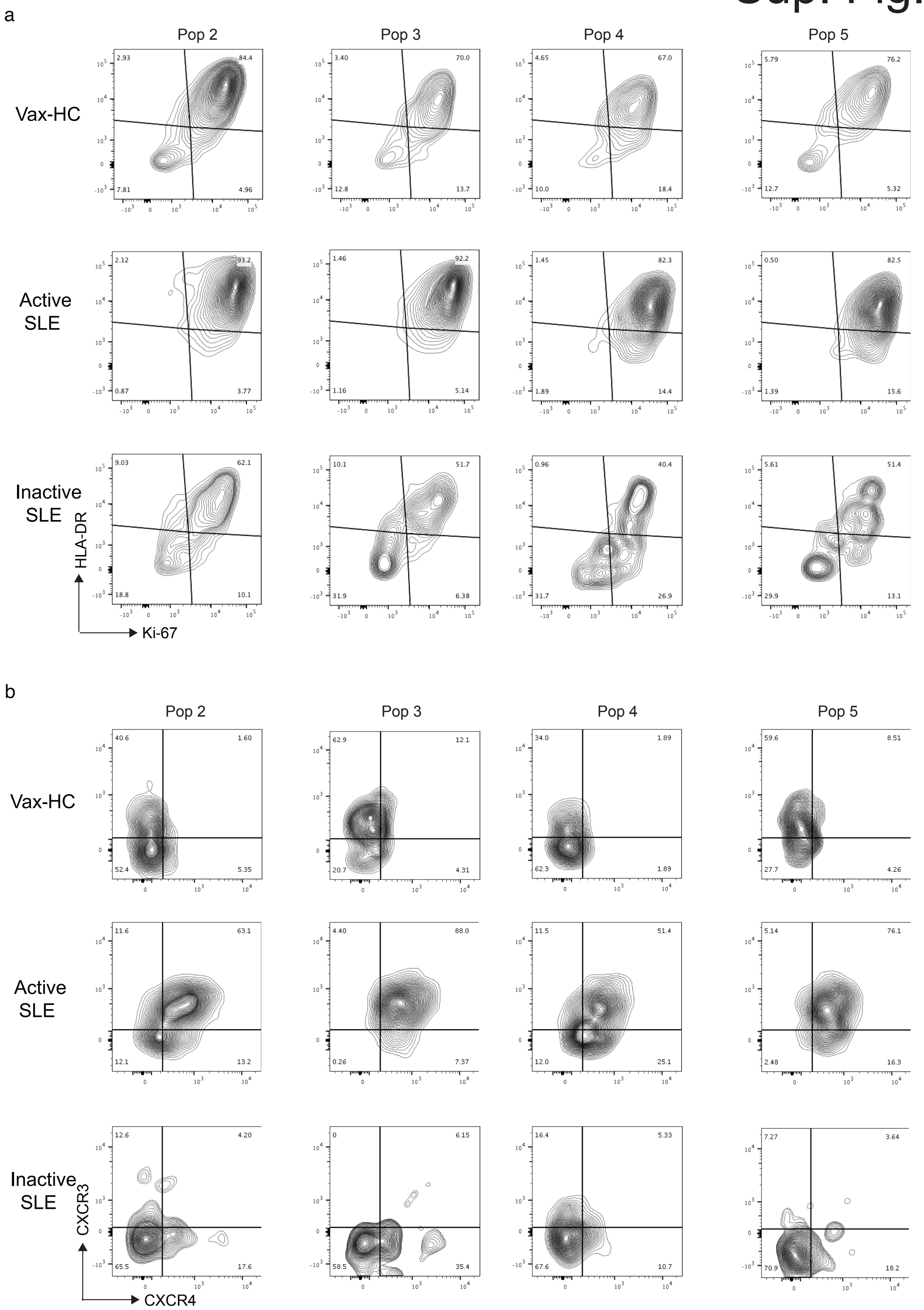

a
Pop 2
Pop 3
Pop 4
Pop 5
Vax-HC
2.93 84.4 7.81 4.96
3.40 70.0 12.8 13.7
4.65 67.0 10.0 18.4
5.79 76.2 12.7 5.32
Active SLE
2.12 93.2 0.87 3.77
1.46 92.2 1.16 5.14
1.45 82.3 1.89 14.4
0.50 82.5 1.39 15.6
Inactive SLE
9.03 62.1 18.8 10.1
10.1 51.7 31.9 6.38
0.96 40.4 31.7 26.9
5.61 51.4 29.9 13.1
HLA-DR
Ki-67
b
Pop 2
Pop 3
Pop 4
Pop 5
Vax-HC
40.6 1.60 52.4 5.35
62.9 12.1 20.7 4.31
34.0 1.89 62.3 1.89
59.6 8.51 27.7 4.26
Active SLE
11.6 63.1 12.1 13.2
4.40 88.0 0.26 7.37
11.5 51.4 12.0 25.1
5.14 76.1 2.48 16.3
Inactive SLE
12.6 4.20 65.5 17.6
0 6.15 58.5 35.4
16.4 5.33 67.6 10.7
7.27 3.64 70.9 18.2
CXCR3
CXCR4

Supplemental Fig. 2. Immune Phenotype of Circulating ASC in Active SLE.

a, Representative co-staining of intracellular Ki-67 and surface HLA-DR on ASC populations from influenza vaccinated heathy subjects on day 7 post immunization, active SLE patients and inactive SLE patients. **b,** Representative co-staining of surface CXCR3 and CXCR4 on ASC populations from influenza vaccinated heathy subjects on day 7 post immunization, active SLE patients and inactive SLE patients.

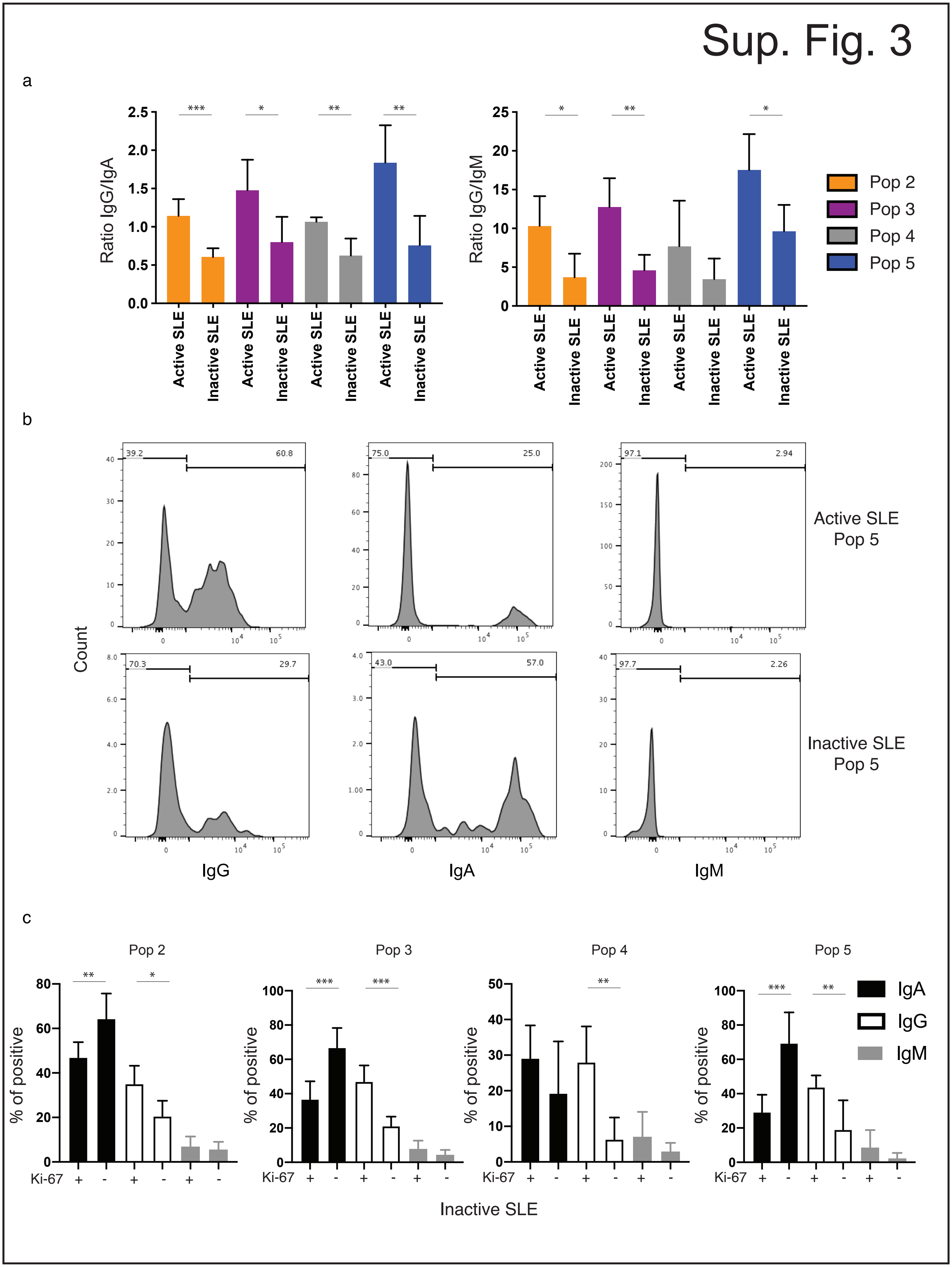

Sup. Fig. 3
a
Ratio IgG/IgA
Ratio IgG/IgM
Active SLE
Inactive SLE
Pop 2
Pop 3
Pop 4
Pop 5
b
Count
Active SLE Pop 5
Inactive SLE Pop 5
IgG
IgA
IgM
c
Pop 2
Pop 3
Pop 4
Pop 5
% of positive
Ki-67
IgA
IgG
IgM
Inactive SLE

Supplemental Fig. 3. IgG-expressing ASC are Enriched in Active SLE Patients.

a, Intracellular staining of peripheral blood ASC from active SLE patients or inactive SLE patients with isotype-specific antibodies. Ratios of IgG/IgA and IgG/IgM in all ASC populations from either active or inactive SLE patients are shown and statistical difference was compared among disease groups within the same population (n = 6). **b,** Representative histograms show the expression of IgG, IgA and IgM in Pop 5 from both active and inactive SLE patients. **c,** The intracellular expression of IgA, IgG, and IgM in proliferating ($Ki\text{-}67^{+}$) versus non-proliferating ($Ki\text{-}67^{-}$) peripheral blood ASC from inactive SLE patients (n = 6). (*$p < 0.05$, **$p < 0.01$, ***$p < 0.001$)

Sup. Table 1

		SLE-1	SLE-2	SLE-3	SLE-4
Cells	Pop 2	60,800	2,830	42,823	18,000
	Pop 3	18,850	1,040	34,783	10,000
	Pop 4	40,920	715	13,252	1,300
	Pop 5	22,000	435	18,574	1,100
Sequences	Pop 2	599	3,520	3,199	2,881
	Pop 3	1,758	6,413	3,837	3,523
	Pop 4	1,790	5,841	2,049	5,053
	Pop 5	2,090	4,982	1,097	3,635
Lineages	Pop 2	527	514	2,292	2,087
	Pop 3	1,305	563	2,816	2,194
	Pop 4	1,446	180	1,296	848
	Pop 5	1,395	231	863	839
D20	Pop 2	51	2	97	132
	Pop 3	79	3	155	106
	Pop 4	118	1	54	9
	Pop 5	60	2	66	11
D50	Pop 2	228	13	743	681
	Pop 3	427	18	941	530
	Pop 4	551	4	311	58
	Pop 5	350	9	341	82

Supplemental Table 1. Summary Statistics of Repertoire Analysis for ASC from Active SLE Samples.

ASC populations from four active SLE patients were used to analyze their clonal repertoire by using next generation sequencing. Numbers of sorted cells, of sequences and of lineages are listed for all populations in each individual. The D_{20} (D_{50}) measure is the number of the largest lineages in a size-ordered list that span 20% (50%) of the sequences.

Sup. Fig. 4

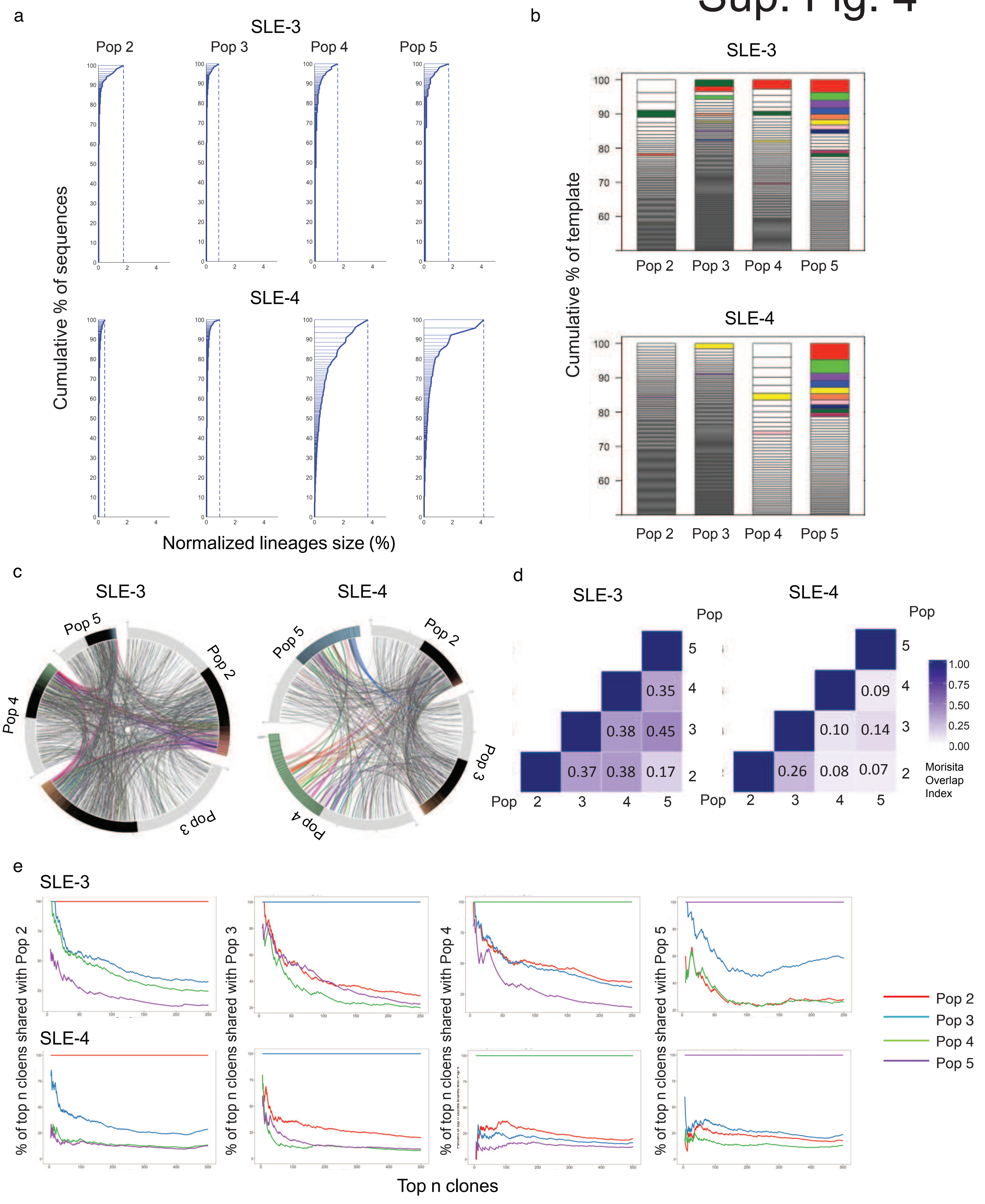

a
SLE-3
Pop 2
Pop 3
Pop 4
Pop 5
SLE-4
Cumulative % of sequences
Normalized lineages size (%)
b
SLE-3
SLE-4
Cumulative % of template
Pop 2
Pop 3
Pop 4
Pop 5
c
SLE-3
SLE-4
d
SLE-3
SLE-4
Pop
0.35
0.38
0.45
0.37
0.38
0.17
0.09
0.10
0.14
0.26
0.08
0.07
Morisita Overlap Index
e
SLE-3
SLE-4
% of top n cloens shared with Pop 2
% of top n cloens shared with Pop 3
% of top n cloens shared with Pop 4
% of top n cloens shared with Pop 5
Top n clones
Pop 2
Pop 3
Pop 4
Pop 5

Supplemental Fig. 4. Heterogeneous SLE ASC Responses Share Common Precursors.

AIRR-seq was used to analyze the clonal repertoire of ASC populations from 2 additional active SLE patients (see Fig. 5). **a,** Clonality of the repertoire in ASC populations is shown by plotting normalized lineage size versus the cumulative percent of sequences. Lineages are size-ranked in descending order along the extent of the y-axis representing 100% of all the sequences. Horizontal lines delineate the individual lineages. **b,** Stacked bar plots demonstrate the diversity of the repertoire by showing descending, size-ranked clones as segments comprising percentages of the total repertoire. The largest 10 clones of the reference population pop 5 are colored, and like-colors in other populations show identical clones in other populations. **c,** Circos plot shows interconnectedness of the ASC populations by plotting the sequences from each population in clonal size-ranked order with the largest clones being the most clockwise portion of each population segment. Lines between ASC populations indicate matched clones between ASC populations. **d,** The Morisita Overlap Index demonstrates the similarity of repertoires in various ASC populations as a value from 0 (no similarity) to 1 (identical repertoire). The color strength is indicative of interconnectivity. **e,** The clonal relatedness of SLE ASC populations is shown by plotting the percent of shared clones with Pop 2, Pop 3, Pop 4, and Pop 5, respectively (y axis), within the top numbers of clones (x axis).

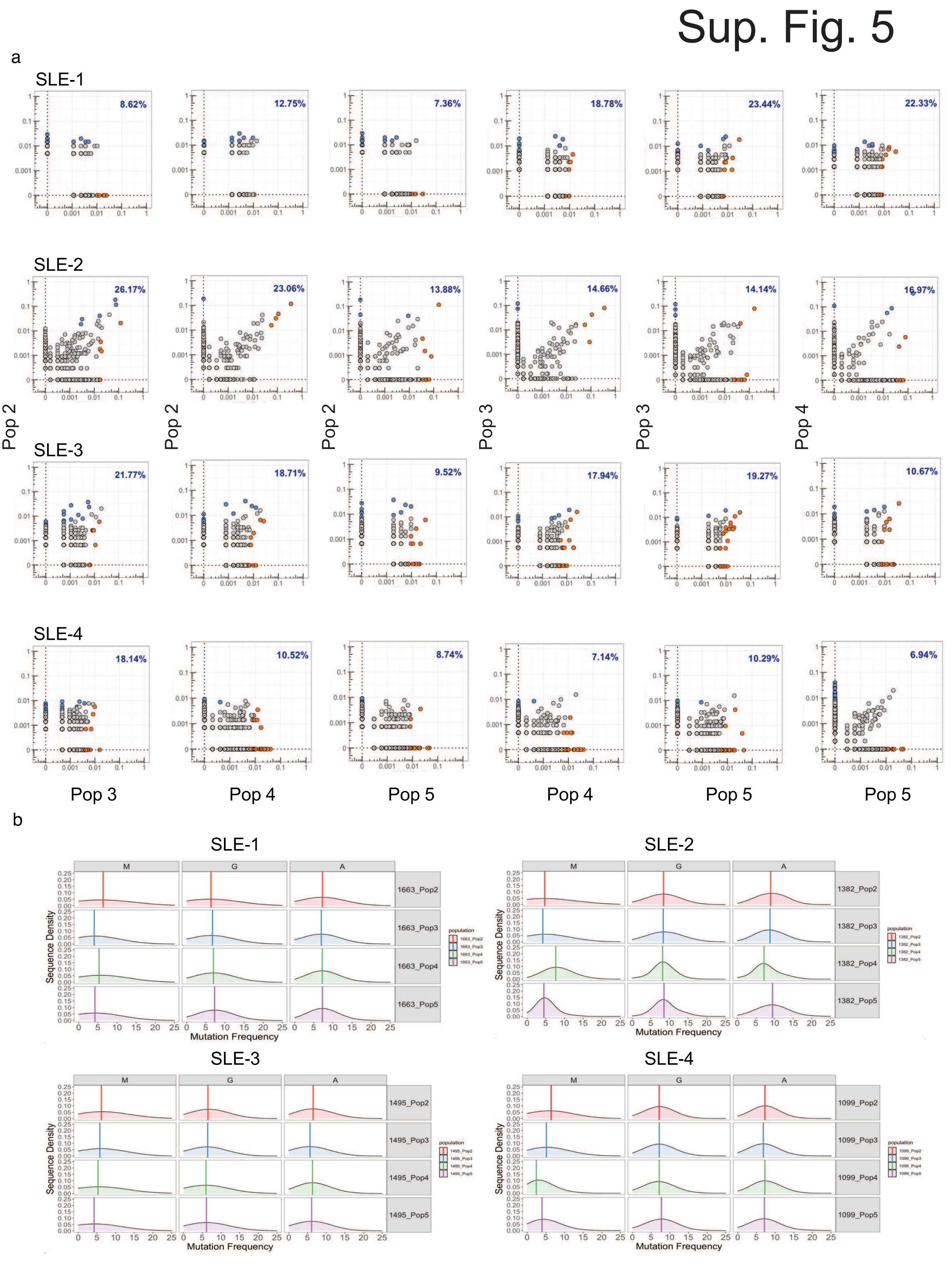
Sup. Fig. 5
a
SLE-1
8.62%
12.75%
7.36%
18.78%
23.44%
22.33%
SLE-2
26.17%
23.06%
13.88%
14.66%
14.14%
16.97%
Pop 2
Pop 2
Pop 2
Pop 3
Pop 3
Pop 4
SLE-3
21.77%
18.71%
9.52%
17.94%
19.27%
10.67%
SLE-4
18.14%
10.52%
8.74%
7.14%
10.29%
6.94%
Pop 3
Pop 4
Pop 5
Pop 4
Pop 5
Pop 5
b
SLE-1
SLE-2
SLE-3
SLE-4
M
G
A
Sequence Density
Mutation Frequency
1663_Pop2
1663_Pop3
1663_Pop4
1663_Pop5
1382_Pop2
1382_Pop3
1382_Pop4
1382_Pop5
1495_Pop2
1495_Pop3
1495_Pop4
1495_Pop5
1099_Pop2
1099_Pop3
1099_Pop4
1099_Pop5
population

Supplemental Fig. 5. Clonal Connectivity and somatic hypermutation analysis of SLE ASC.

a, Scatter plots display clone frequencies of two populations that are shared (in the middle) or unique (on the axis). Clone frequencies that are significantly different between the populations are colored with orange (increased on the X axis population) or blue (increased on the Y axis population). Percent of clones that were found to be shared between populations is shown in the top right. **b**, Somatic hypermutation frequencies of each ASC population from four active SLE patients. The curve shows the density of sequences at each mutation frequency and the vertical line is the average for the ASC population expressing either IgM, IgG or IgA.

Sup. Fig. 6

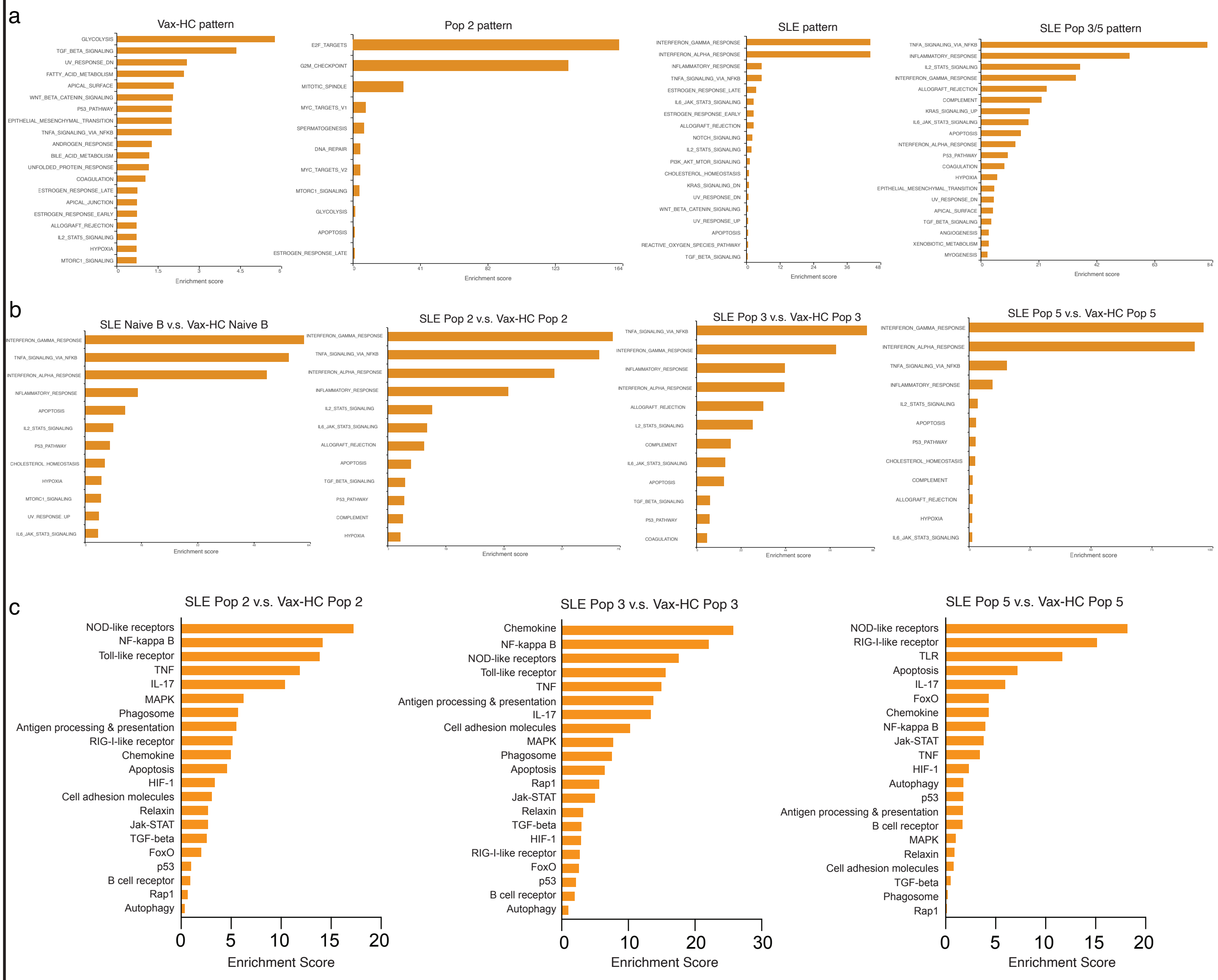

a
Vax-HC pattern
GLYCOLYSIS
TGF_BETA_SIGNALING
UV_RESPONSE_DN
FATTY_ACID_METABOLISM
APICAL_SURFACE
WNT_BETA_CATENIN_SIGNALING
P53_PATHWAY
EPITHELIAL_MESENCHYMAL_TRANSITION
TNFA_SIGNALING_VIA_NFKB
ANDROGEN_RESPONSE
BILE_ACID_METABOLISM
UNFOLDED_PROTEIN_RESPONSE
COAGULATION
ESTROGEN_RESPONSE_LATE
APICAL_JUNCTION
ESTROGEN_RESPONSE_EARLY
ALLOGRAFT_REJECTION
IL2_STAT5_SIGNALING
HYPOXIA
MTORC1_SIGNALING
Enrichment score
Pop 2 pattern
E2F_TARGETS
G2M_CHECKPOINT
MITOTIC_SPINDLE
MYC_TARGETS_V1
SPERMATOGENESIS
DNA_REPAIR
MYC_TARGETS_V2
MTORC1_SIGNALING
GLYCOLYSIS
APOPTOSIS
ESTROGEN_RESPONSE_LATE
Enrichment score
SLE pattern
INTERFERON_GAMMA_RESPONSE
INTERFERON_ALPHA_RESPONSE
INFLAMMATORY_RESPONSE
TNFA_SIGNALING_VIA_NFKB
ESTROGEN_RESPONSE_LATE
IL6_JAK_STAT3_SIGNALING
ESTROGEN_RESPONSE_EARLY
ALLOGRAFT_REJECTION
NOTCH_SIGNALING
IL2_STAT5_SIGNALING
PI3K_AKT_MTOR_SIGNALING
CHOLESTEROL_HOMEOSTASIS
KRAS_SIGNALING_DN
UV_RESPONSE_DN
WNT_BETA_CATENIN_SIGNALING
UV_RESPONSE_UP
APOPTOSIS
REACTIVE_OXYGEN_SPECIES_PATHWAY
TGF_BETA_SIGNALING
Enrichment score
SLE Pop 3/5 pattern
TNFA_SIGNALING_VIA_NFKB
INFLAMMATORY_RESPONSE
IL2_STAT5_SIGNALING
INTERFERON_GAMMA_RESPONSE
ALLOGRAFT_REJECTION
COMPLEMENT
KRAS_SIGNALING_UP
IL6_JAK_STAT3_SIGNALING
APOPTOSIS
INTERFERON_ALPHA_RESPONSE
P53_PATHWAY
COAGULATION
HYPOXIA
EPITHELIAL_MESENCHYMAL_TRANSITION
UV_RESPONSE_DN
APICAL_SURFACE
TGF_BETA_SIGNALING
ANGIOGENESIS
XENOBIOTIC_METABOLISM
MYOGENESIS
Enrichment score
b
SLE Naive B v.s. Vax-HC Naive B
INTERFERON_GAMMA_RESPONSE
TNFA_SIGNALING_VIA_NFKB
INTERFERON_ALPHA_RESPONSE
INFLAMMATORY_RESPONSE
APOPTOSIS
IL2_STAT5_SIGNALING
P53_PATHWAY
CHOLESTEROL_HOMEOSTASIS
HYPOXIA
MTORC1_SIGNALING
UV_RESPONSE_UP
IL6_JAK_STAT3_SIGNALING
Enrichment score
SLE Pop 2 v.s. Vax-HC Pop 2
INTERFERON_GAMMA_RESPONSE
TNFA_SIGNALING_VIA_NFKB
INTERFERON_ALPHA_RESPONSE
INFLAMMATORY_RESPONSE
IL2_STAT5_SIGNALING
IL6_JAK_STAT3_SIGNALING
ALLOGRAFT_REJECTION
APOPTOSIS
TGF_BETA_SIGNALING
P53_PATHWAY
COMPLEMENT
HYPOXIA
Enrichment score
SLE Pop 3 v.s. Vax-HC Pop 3
TNFA_SIGNALING_VIA_NFKB
INTERFERON_GAMMA_RESPONSE
INFLAMMATORY_RESPONSE
INTERFERON_ALPHA_RESPONSE
ALLOGRAFT_REJECTION
IL2_STAT5_SIGNALING
COMPLEMENT
IL6_JAK_STAT3_SIGNALING
APOPTOSIS
TGF_BETA_SIGNALING
P53_PATHWAY
COAGULATION
Enrichment score
SLE Pop 5 v.s. Vax-HC Pop 5
INTERFERON_GAMMA_RESPONSE
INTERFERON_ALPHA_RESPONSE
TNFA_SIGNALING_VIA_NFKB
INFLAMMATORY_RESPONSE
IL2_STAT5_SIGNALING
APOPTOSIS
P53_PATHWAY
CHOLESTEROL_HOMEOSTASIS
COMPLEMENT
ALLOGRAFT_REJECTION
HYPOXIA
IL6_JAK_STAT3_SIGNALING
Enrichment score
c
SLE Pop 2 v.s. Vax-HC Pop 2
NOD-like receptors
NF-kappa B
Toll-like receptor
TNF
IL-17
MAPK
Phagosome
Antigen processing & presentation
RIG-I-like receptor
Chemokine
Apoptosis
HIF-1
Cell adhesion molecules
Relaxin
Jak-STAT
TGF-beta
FoxO
p53
B cell receptor
Rap1
Autophagy
0 5 10 15 20
Enrichment Score
SLE Pop 3 v.s. Vax-HC Pop 3
Chemokine
NF-kappa B
NOD-like receptors
Toll-like receptor
TNF
Antigen processing & presentation
IL-17
Cell adhesion molecules
MAPK
Phagosome
Apoptosis
Rap1
Jak-STAT
Relaxin
TGF-beta
HIF-1
RIG-I-like receptor
FoxO
p53
B cell receptor
Autophagy
0 10 20 30
Enrichment Score
SLE Pop 5 v.s. Vax-HC Pop 5
NOD-like receptors
RIG-I-like receptor
TLR
Apoptosis
IL-17
FoxO
Chemokine
NF-kappa B
Jak-STAT
TNF
HIF-1
Autophagy
p53
Antigen processing & presentation
B cell receptor
MAPK
Relaxin
Cell adhesion molecules
TGF-beta
Phagosome
Rap1
0 5 10 15 20
Enrichment Score

Supplemental Fig. 6. SLE ASC Express Abnormal Pathways.

a, GSEA pathways analysis shows the top pathways that are modulated in the four patterns identified in Fig. 6d. **b**, GSEA pathways analysis identifies the top pathways that are upregulated in ASC populations and naïve B cells from active SLE patients versus vaccinated healthy subjects. **c**, KEGG pathway analysis of DEG identified from the SLE and post-vax HC for each ASC populations.
